# Seasonal Elevational Migration Shapes Temperate Bird Community in the Gyirong Valley, Central Himalayas

**DOI:** 10.3390/biology15020138

**Published:** 2026-01-13

**Authors:** Huaiming Jin, Shuqing Zhao, Zhifeng Ding, Yongbing Yang, Gang Song, Shuaishuai Huang, Ruojin Liu, Shengling Zhou, Le Yang, Yonghong Zhou

**Affiliations:** 1 Key Laboratory of Biodiversity and Environment on the Qinghai-Tibetan Plateau, Ministry of Education, Xizang University, Lhasa 850099, China; jinhuaiming2025@163.com (H.J.); z05232025@163.com (S.Z.);; 2 School of Ecology and Environment, Xizang University, Lhasa 850099, China; 3 Yani Wetland Ecosystem Positioning Observation and Research Station, Xizang, Lhasa 850099, China; 4 Guangdong Key Laboratory of Animal Conservation and Resource Utilization, Institute of Zoology, Guangdong Academy of Sciences, Guangzhou 510260, China; dingzhf@163.com (Z.D.); zsl13518916139@163.com (S.Z.); 5 Key Laboratory of Biological Resources and Biosafety, Institute of Plateau Biology Research of Xizang Autonomous Region, Lahsa 850000, China; 18270292589@139.com; 6 Key Laboratory of Animal Biodiversity Conservation and Integrated Pest Management, Institute of Zoology, Chinese Academy of Sciences, Beijing 100101, China; songgang@ioz.ac.cn

**Keywords:** seasonal migration, climate adaptation, community assembly dynamics, morphological adaptation, niche separation, temporal beta diversity

## Abstract

Understanding the mechanisms underlying seasonal community dynamics is important for predicting biodiversity responses to environmental fluctuations, enhancing ecological forecasting, and informing conservation strategies. In this study, we use standard transect and mist netting methods investigated seasonal altitudinal migration patterns of montane bird species in the Gyirong Valley, Central Himalayas. Our results showed four distinct altitudinal migration patterns among montane bird species: no shift, downslope shift, upslope shift, and contraction to mid-elevation zones. Species with smaller body weight and higher ratios of wing length, tail length, and tarsus length to body weight tended to migrate to lower elevations. Insectivorous birds exhibited a collective downslope shift, while omnivorous birds showed a wider range of migratory responses to seasonal variation. Migratory behavior was found to dynamically modulate the association between phenotypic traits and habitat preferences. During the breeding season, species (70.44%) and functional turnover (80.02%) dominated, while in the non-breeding season, nestedness significantly contributed to species (49.37%) and functional diversity (38.09%). In addition, migration can disrupt the direct influence of environmental variables on biodiversity patterns, providing important insights for montane biodiversity conservation under climate change. Our results highlight the critical need to safeguard low-elevation winter habitats and create dynamic protected areas to aid bird conservation amidst climate change.

## Simple Summary

This study explores how birds in the Gyirong Valley of the central Himalayas adapt to seasonal changes by moving up and down mountain slopes. We aimed to understand their migration patterns and how this affects bird communities. Our findings show that birds exhibit four main patterns: some do not migrate, others move downhill, some uphill, and a few contraction to mid-elevation. Smaller birds with longer wings and tails relative to their weight tend to migrate downhill to escape cold weather. Insect-eating birds often move downhill together, while birds that eat both plants and insects show more flexible responses. Seasonal migration reshapes how bird traits relate to their distribution, leading to significant community changes during breeding season and greater stability in non-breeding periods. These results support the idea that birds separate their niches seasonally to reduce competition and cope with environmental stress. This research is valuable for conservation efforts, emphasizing the need to protect low-elevation winter habitats and create dynamic protected areas to help birds survive climate change, ultimately benefiting biodiversity management.

## 1. Introduction

The spatiotemporal dynamics of bird communities are a core issue in ecological research [1,2]. The distribution changes in species across temporal and spatial scales not only shape the diversity patterns of the earth’s living systems but also serve as a key driving force for the maintenance and evolution of ecosystem functions [3]. Understanding the temporal and spatial distribution patterns of mountain birds provides scientific guidance for the formulation of conservation strategies for mountain birds. Although the spatial distribution patterns of mountain birds have been widely studied [4,5], research on the seasonal dynamics of mountain bird community structure and their driving factors is still insufficient.

Several studies have explored the patterns of elevation migration of mountain birds in response to seasonal changes, finding that mountain birds have patterns of upslope shift, downslope shift, and no shift [6,7,8]. This ecological strategy leads to species turnover and changes in diversity along the elevation gradient [6]. Importantly, elevation migration has a dual impact on community assembly: it promotes genetic exchange and niche differentiation through seasonal species turnover, while potentially intensifying intraspecific and interspecific competition through niche compression [6]. However, previous studies have primarily focused on community structure within a single season, often overlooking the spatiotemporal dynamic driven by migratory behavior [7,9,10]. There remains a gap on that how seasonal migration systematically reshapes community composition, functional traits, and assembly mechanisms, and the analysis of seasonal altitudinal distribution changes in mountain birds provides a new perspective for understanding the seasonal dynamics of mountain bird community structure.

Ecological traits are important factors that influence bird migratory behavior. Diet, body size, and habitat preference are key factors that affect birds’ altitudinal migration [6,7,8]. In addition, wing length, tail length, and tarsus length are closely related to birds’ migration distance, flight stability, and habitat adaptation [11,12,13]. Insectivorous birds, for example, tend to migrate downward to cope with seasonal resource fluctuations, while omnivorous birds exhibit phenotypic plasticity, balancing resource utilization efficiency and risk [6,7,8]. Additionally, migratory birds generally possess longer wings and lighter body weights, traits that enhance flight efficiency [6,7,14]. So we propose the hypothesis of “seasonal niche separation”; birds maintain energy metabolic efficiency and avoid interspecific competition by seasonally adjusting their altitudinal distribution.

In mountainous ecosystems, dramatic fluctuations in seasonal environmental gradients, such as temperature and precipitation, drive the widespread occurrence of elevation migration in birds [15]. Different birds respond differently to environmental changes [16,17,18], and elevation migration may lead to seasonal reallocation of resources, thereby altering the distribution patterns of mountain birds and the relationship between environmental factors and bird diversity. Beta diversity is influenced by environmental filtering and species competition [19,20], and the temporal beta diversity can reflect the migration patterns of species. When turnover is dominant, it indicates that species exhibit significant migration across different habitats or elevational gradients; when nestedness is dominant, it suggests that the range of species migration is relatively small, and species migration between different elevations or habitats is restricted.

Located in the central section of the Himalayas, the Gyirong Valley 28°15′–29° N, 85°6′–85°41′ E provides an ideal research platform for solving the aforementioned puzzles. As an important passage for the northward movement of warm and humid air from the Indian Ocean [21], this region features a complete mountain vertical belt spectrum: a continuous climatic gradient from the subtropical mountains (1700 m) to the alpine frigid climate (5500 m), which has given rise to a variety of bird species and rich flora and fauna [22]. Its unique geographical location and climatic heterogeneity make it a natural laboratory for studying the ecology of seasonal migration. It is worth noting that, although previous studies have revealed the pattern of community assembly dominated by environmental filtering during the breeding season in this region [20], there is still a lack of in depth understanding of the association mechanisms between non-breeding season bird migration behavior and community dynamics. In particular, the application of the three-dimensional β-diversity decomposition model in seasonal analysis remains a blank, which restricts our understanding that how migratory behavior regulates the relationship between environmental factors and diversity.

This study aims to reveal the dynamic regulatory mechanisms of community assembly driven by seasonal altitude migration of birds in the Gyirong Valley through an integrated multi-seasonal analysis. Specifically, it focuses on the following scientific questions: (1) What are the migratory patterns of temperate birds in the Himalayas and their associations with ecological traits? (2) How does seasonal migration drive changes in community composition through the reconfiguration of trait-environment associations? (3) Does seasonal migration lead to seasonal reversals in community assembly mechanisms (species turnover vs. nestedness)? Based on the three-dimensional β-diversity decomposition model, we propose the “seasonal niche separation” hypothesis: birds adjust their altitudinal distribution seasonally to maintain metabolic efficiency while avoiding interspecific competition, thereby driving systematic reorganization of community structure between the breeding and non-breeding seasons.

## 2. Materials and Methods

### 2.1. Study Area

The Gyirong Valley is one of the five major valleys on the southern slope of the Himalayas, ranging from 1700 m at the lowest point to 5500 m at the highest point, and forms a distinct vertical climatic belt spectrum [22]. Influenced by the Indian Ocean monsoon, the annual average precipitation in the Gyirong Valley ranges from 1000 to 3000 mm [23], with precipitation primarily concentrated from November to March (in the form of frost and snow) and from June to August (in the form of rainfall).

There is a significant vertical climatic-vegetation differentiation in Gyirong Valley, with climatic zones distributed as follows: the Mountainous Subtropical Zone (1700–2500 m), the Mountainous Warm Temperate Zone (2500–3300 m), the Mountainous Cold Temperate Zone (3300–3900 m), the Subalpine Frigid Zone (3900–4700 m), and the Alpine Frigid Zone (4700–5500 m). The corresponding vegetation types include evergreen broadleaf forest (1700–2500 m), coniferous and broadleaf mixed forest (2500–3300 m), dark coniferous forest (3300–3900 m), shrub and grass (3900–4700 m), and alpine tundra with sparse grass (4700–5500 m) [8,22]. This climatic and vegetative heterogeneity provides suitable habitats and migratory spaces for birds with different ecological niches, making it an ideal region for studying the mechanisms of seasonal community dynamics. Human activities are mainly concentrated below mid-elevations, in the forms of tourism, transportation, and agriculture [24].

### 2.2. Data Collection

Our study focused on the mid-altitude gradient of 2800–4000 m, as this altitudinal range encompasses the overlap and transition zone of population distribution during both the breeding and non-breeding seasons, making it a core activity area for birds’ seasonal vertical migration. From May to July and November to December 2024, birds were surveyed using the standard transect method [25] (pp. 56–68) and mist-netting [26], and the morphological traits (weight, body length, wing length, tail length, tarsus length, and bill length) of captured birds were measured. These surveys were conducted across four elevation gradient bands (2800–4000 m), established using a 300 m interval [6,27,28], with five transects (total length ~8 km) set up within each band. Specifically, the five transects per band covered a variety of habitats, including coniferous forests, broadleaf forests, mixed forests, agricultural fields, shrublands, and grasslands. Each transect was surveyed twice: once during the breeding season and once during the non-breeding season. The survey times were selected to cover the 4 h after sunrise and the 3 h before sunset, corresponding to the peak activity periods for birds, with observations conducted at a walking speed of 2 km/h. In dense vegetation habitats (e.g., coniferous forests, broadleaf forests, and mixed forests), the survey area extended 25 m on each side of the transect, while in open habitats (e.g., agricultural fields and grasslands), it extended 50 m [29].

**Figure 1:**
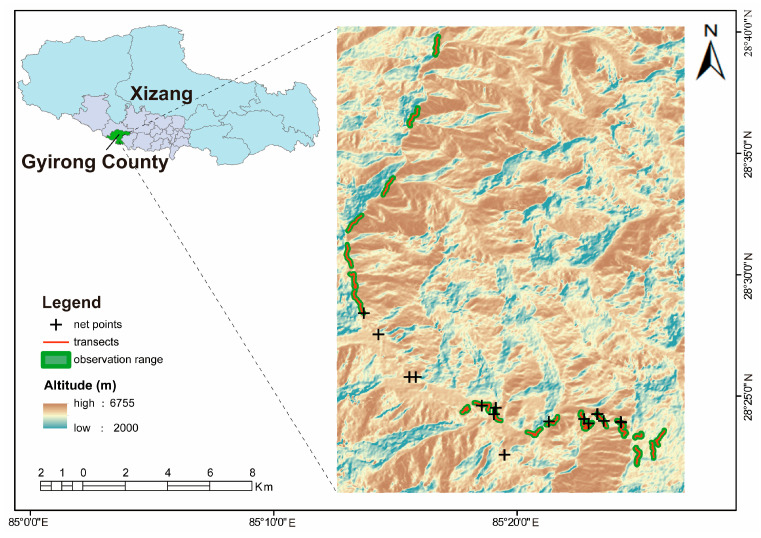
Distribution map of transects and mist-netting sites in the Gyirong Valley.

Mist nets were placed in woodland and shrub areas to enhance the capture rate of bird species, as these habitats are rich in bird diversity based on our field observations. A total of 14 netting sites were established (three to four mist nets were set up in each elevational band Figure 1), with 2 to 3 mist nets installed at each site. We operated each mist net for a one-day session and checked the nets every hour. The morphological traits of the captured birds (including body weight, body length, wing length, tail length, tarsus length, and bill length) were measured promptly. The birds were then marked by clipping a small notch in the tail feather using scissors, and released back into their original habitat.

### 2.3. Data Analysis

(1) Classification of migration patterns and trait-migration relationships

To ensure the reliability of the results, we only retained species that were recorded during both the breeding and non-breeding seasons and excluded those that fly at high altitudes (we excluded highly aerial species, including swifts and raptors, as the elevational ranges of these species might be inaccurate due to elevational differences between surveyors and birds when detected) for migration pattern analysis. These treatments resulted in 35 species for further analyses. We determined the migration patterns of birds by calculating the weighted mean center of species’ altitude distribution M = Σ_(m,n)_E_i_ × P_Ai_, and combining the upper and lower limits of species’ altitude distribution [6,7], where m and n are the range limits of species A, E_i_ is the altitude (m) of site i, and P_Ai_ is the proportion of individuals of species A at site i in its total individuals recorded along the whole gradient: (1) No-shift type: (M_2_ − M_1_ = 0), and M_2_ and M_1_ are in the same altitude zone; (2) Downslope shift type: (M_2_ − M_1_ < 0), and M_2_ is in a lower altitude zone; (3) Upslope shift type: (M_2_ − M_1_ > 0), and M_2_ is in a higher altitude zone; (4) Contraction to mid-elevation type: (M_2_ − M_1_ = 0, and both the upper and lower limits of the species’ altitude distribution during the non-breeding season contract towards mid-elevation). M_2_ is the weighted mean center altitude during the non-breeding season for a certain bird species, and M_1_ is the weighted mean center altitude during the breeding season for that species.

Body mass is an index for dispersal ability in birds, with larger size assumed to indicate greater power [30]. To explore the impact of morphological traits on the seasonal migration of mountain birds, we used the Wilcoxon rank-sum test [31] to conduct a difference analysis on key ratios such as wing length to body weight (wl/wt), tail length to body weight (tl/wt), tarsus length to body weight (tar/wt), and body weight (weight was normalized) between the downslope shift group and the no shift group, with a significance level set at *p* = 0.05 [32,33].

To explore the preference of seasonal altitudinal migration patterns among different phylogenetic clades and diet groups of birds, we conducted phylogenetic cluster analysis based on the Linnaean classification system for the selected birds and combined it with diet data (such as insectivorous, omnivorous, etc.) [34] to investigate the preference in migratory strategies among different clades and diet groups.

(2) Trait-distribution relationships and beta temporal diversity analysis

Seasonal migration can alter the impact of morphological traits on the distribution of mountain birds. To identify key factors that contribute to the altitudinal distribution of birds during the breeding and non-breeding seasons, we applied the random forest (RF) algorithm, as it does not require strict assumptions about the data. In the RF model, the response variable was bird distribution pattern, while the predictor variables were bird morphological traits. Morphological traits were removed with multicollinearity using Variance Inflation Factor (VIF > 10) [35]. To assess the relative importance of each morphological trait, we used the average of the percentage increase in mean squared error (%IncMSE) within RF models built after permuting the values of the considered trait, with a higher importance value suggesting that trait to be more influential than others in explaining the altitudinal distribution of birds. Random forest regression models were built using the “randomForest 4.0-0” package [36] in the R v4.2.1 software [37].

Seasonal migration may alter the contributions of turnover and nestedness to β-diversity. To explore the effects of environmental filtering and species competition on β-diversity in different seasons, we conducted taxonomic, functional, and phylogenetic beta diversity decomposition following Baselga’s method [38].

(3) Community structure driven by seasonal environmental factors

Migratory behavior may alter the influence of environmental factors on the structure of bird communities. To understand the role of environmental factors in the seasonal dynamics of bird community structure, key environmental factors such as altitude, NDVI (Normalized Difference Vegetation Index), monthly mean temperature, and monthly mean precipitation were extracted based on previous research results [23,27]. Variance Inflation Factor (VIF) [35] was used to test and control for multicollinearity among environmental variables. The following studies were conducted: CCA was used to determine the importance of environmental factors to bird communities, and correlation analysis was combined to further compare the changes in the strength of environmental factors between breeding and non-breeding seasons, revealing the buffering mechanism of migratory behavior against environmental stress [39].

## 3. Results

### 3.1. Seasonal Bird Migration Patterns and Influencing Factors

**Figure 2:**
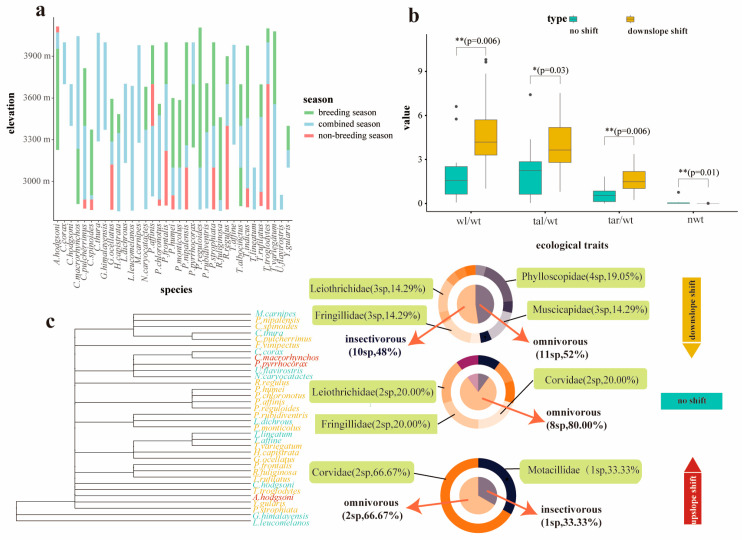
Seasonal migration patterns of birds and their influencing factors. (**a**) shows the bird migration patterns (species name are represented by the genus initial and specific epithet). (**b**) illustrates the ecological trait differences among birds with different migration patterns (wl: wing length; wt: weight; tar: tarsus; nwt: normalized body weight), ”*” indicates *p* < 0.05, “**” indicates *p* < 0.01. (**c**) displays the classification of different bird migration patterns and their dietary compositions.

Thirty-five bird species were recorded in both the breeding and non-breeding seasons, exhibiting three primary patterns of elevational migration: no shift (10 species), downslope shift (21 species), and upslope shift (3 species). In addition, one species—the *Turdus albocinctus* showed a range contraction toward the mid-elevation zone (e.g., 3200–3600 m) (Figure 2a). Morphological traits were significantly associated with elevational migration strategies (Figure 2b). Specifically, birds exhibiting downslope shifts had significantly higher wing length-to-body weight (wl/wt), tail length-to-body weight (tl/wt), and tarsus length-to-body weight (tar/wt) ratios compared to species with no shifts (*p* < 0.05). Additionally, smaller-bodied birds were more likely to exhibit downslope shifts in response to decreasing temperatures (*p* = 0.01).

Cluster analysis revealed distinct taxonomic patterns associated with elevational migration strategies (Figure 2c). Among the downslope-shifting species, Phylloscopidae was the most represented family (4 species, 19.05%), followed by Fringillidae, Leiothrichidae, and Muscicapidae, each contributing 3 species (14.29%). In contrast, the no-shift group was primarily composed of species from Corvidae, Fringillidae, and Leiothrichidae, with 2 species within each family (20%). Upslope shifts were predominantly observed in the Corvidae family (2 species, 66.67%). From a dietary perspective, insectivorous species (n = 10) predominantly exhibited downslope shifts during the non-breeding season, while omnivorous species exhibited more variable migration patterns, including downslope shifts (11 species), no shifts (8 species), and upslope shifts (2 species) (Figure 2c).

### 3.2. Migration Modulates Morphology–Distribution Associations

**Figure 3:**
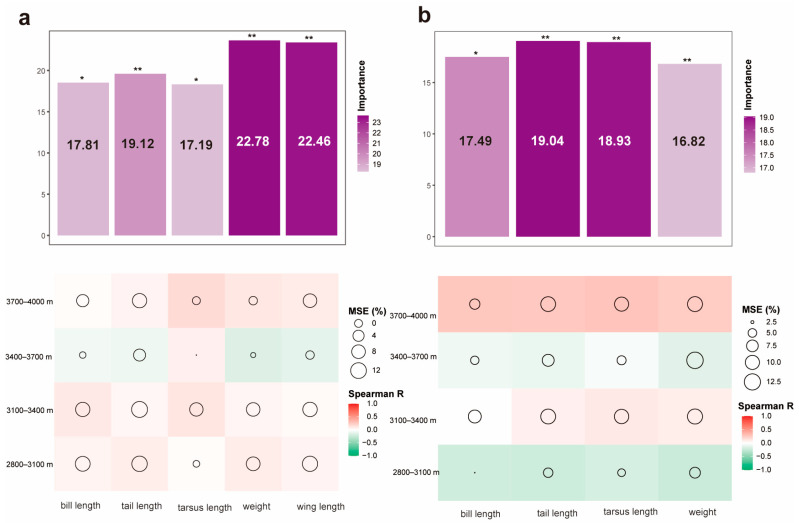
The relationship between bird morphological traits and population distribution reshaped by migration behavior. Panel (**a**) represents the relationship during the breeding season, and panel, ”*” indicates *p* < 0.05, “**” indicates *p* < 0.01. (**b**) represents the relationship during the non-breeding season, ”*” indicates *p* < 0.05, “**” indicates *p* < 0.01.

Based on the results of the variance inflation factor analysis, body length was removed in the breeding season, while both body length and wing length were removed in the non-breeding season. The importance of morphological traits accounted for 99.36% and 72.28% of the variation in bird species distribution along elevation during the breeding and non-breeding seasons, respectively. Larger-bodied birds with longer bills, tails, and tarsus, as well as greater body weight and wing length were concentrated at lower (2800–3400 m) and higher (3700–4000 m) elevations, while smaller bodied-sized birds dominated mid-elevations (3400–3700 m). Seasonal shifts in these patterns indicate that elevational migration mediates the relationship between morphology traits and distribution dynamics (Figure 3).

### 3.3. Seasonal Migration Effects on β-Diversity and Environmental Drivers

**Figure 4:**
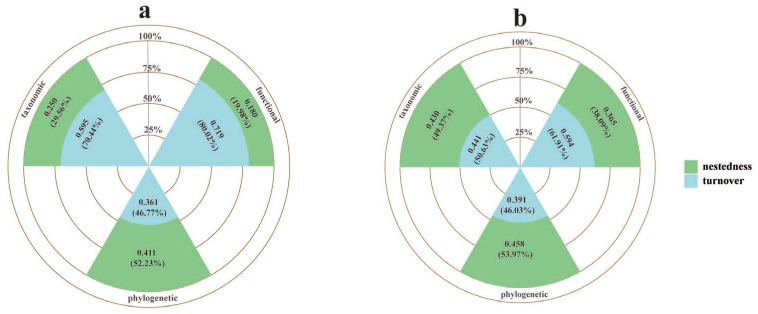
The impact of seasonal bird migration on β-diversity decomposition ((**a**) represents the β-diversity decomposition results for the breeding season, and (**b**) represents the β-diversity decomposition results for the non-breeding season).

Seasonal shifts in β-diversity components revealed contrasting community structure. During the breeding season, species turnover (70.44%) and functional turnover (80.02%) were the dominated components, while the non-breeding season showed increased contributions from nestedness to both taxonomic (49.37%) and functional β-diversity (38.09%). Phylogenetic β-diversity was consistently dominated by nestedness throughout the year, with breeding and non-breeding season contributions of 52.23% and 53.97%, respectively (Figure 4).

**Figure 5:**
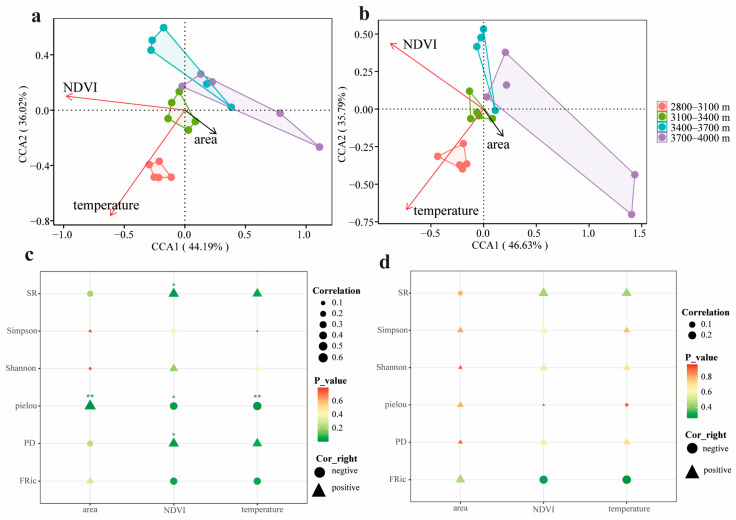
The impact of seasonal bird migration on the relationship between diversity and environmental factors ((**a**) shows the results of CCA for the breeding season, and (**b**) shows the results for the non-breeding season. (**c**) represents the impact of environmental factors on bird diversity during the breeding season, and (**d**) represents the relationship between environmental factors and bird diversity during the non-breeding season).

The results of Canonical Correspondence Analysis (CCA) indicate that the similarity among bird species is higher in the 2800–3400 m elevation zone, while differences in bird composition among transects are greater in the 3400–4000 m elevation zone. During both breeding and non-breeding seasons, the environmental factors influencing bird community composition are NDVI and temperature (*p* < 0.01), while area has a relatively small effect (*p* > 0.05) (Figure 5a,b). Environmental drivers also varied seasonally. During the breeding season, area positively affected community evenness (*p* < 0.01), NDVI was positively associated with species abundance and phylogenetic diversity (*p* < 0.05), but negatively with evenness (*p* < 0.05), and increasing temperature reduced evenness (*p* < 0.01). In the non-breeding season, environmental effects were largely non-significant (Figure 5c,d), suggesting that migratory behavior buffers bird communities against environmental fluctuations and promotes annual-scale stability in montane ecosystems.

## 4. Discussion

In this study, we examine the migratory dynamics of temperate bird communities in the central Himalayas during breeding and non-breeding seasons. Our results show that birds exhibit adaptive morphological traits in response to migration. Downslope migrants have larger wing-to-body, tail-to-body, and tarsus-to-body ratios, as well as smaller body weight, which helps them cope with low temperature. Diet also shapes migration strategies, with insectivores predominantly migrating downslope, while omnivores exhibit more flexible patterns. Seasonal migration alters community structure, with species turnover dominating in the breeding season and increased nestedness in the non-breeding season. “Seasonal niche separation” refers to a phenomenon where species adjust their niche dimensions (including spatial distribution, food selection, activity rhythms, etc.) in response to seasonal environmental changes (such as temperature, food availability, habitat conditions, etc.), so as to reduce interspecific competition, adapt to environmental stress, and maximize survival and reproduction success rates [40]. The study provides evidence that elevational migration mediates ecological niche construction and buffers environmental stress, supporting the “seasonal niche separation” hypothesis [41,42].

### 4.1. Migration Patterns and Ecological Adaptation Mechanisms

The four seasonal migration patterns observed in this study (no shift, downslope shift, upslope shift, and contracting to mid-elevation zone) (Figure 2a) are largely consistent with previous findings from the Hengduan Mountains and central Himalayas regions [6,7,8]. These results demonstrate the broad applicability of four migration patterns for montane birds. Specifically, birds exhibiting downslope shifts show significant morphological adaptations, including higher wing length to body weight, and tail length to body weight ratios, as well as smaller body weight, possibly related to coping with cold stress (*p* = 0.01). These patterns align with Bergmann’s rule, which suggests that larger individuals have a survival advantage in colder environments, whereas smaller individuals, unable to tolerate the cold, are forced to migrate downslope [43,44,45]. Furthermore, our findings support the “flight efficiency adaptation hypothesis”, which posits that smaller body weight and longer wingspans help reduce energy expenditure, thus enabling birds to better cope with cold environments [14,46]. This is further emphasized by the significant tendency for smaller individuals to migrate downslope (*p* = 0.01) (Figure 2b), underscoring the critical role of energy-saving strategies in seasonal adaptation. In contrast, *Turdus albocinctus* exhibits a distinct migratory pattern, contracting to the mid-elevation zone (3100–3400 m) during winter, unlike the typical strategy of breeding at high elevations and overwintering at lower ones [6,8]. This unique behavior may be linked to the availability of winter pastures at this altitude. Unlike long-distance migrants, many forest birds in the region shift to human-dominated non-forest habitats at lower elevations, suggesting that birds optimize resource access by adjusting their spatial distribution rather than migrating solely based on distance [47,48,49]. Furthermore, mammals and insects also exhibit similar altitude migration phenomena [50,51].

When considering dietary habits, we found that downslope shifts were more pronounced in insectivorous birds (a total of 10 species from Muscicapidae and Phylloscopidae) during the non-breeding season, consistent with previous findings [52,53,54]. This is likely due to their strong dependence on energy-rich insect prey at lower elevations, where terrestrial insect richness is particularly high, as shown by a recent global meta-analysis [55]. In contrast, omnivorous birds (e.g., Corvidae, Fringillidae, Leiothrichidae) exhibited a broader range of migratory strategies: 11 species shifted downslope, 8 remained stationary, and 2 shifted upslope—demonstrating greater phenotypic plasticity (Figure 2c). This flexibility, potentially facilitated by dietary adjustments such as incorporating more plant-based foods during non-breeding season [56], may offer an adaptive advantage under changing environmental conditions. These contrasting patterns highlight the significant role of dietary traits in shaping elevational migration strategies and underscore clear niche differentiation in energy acquisition.

### 4.2. Seasonal Restructuring of Morphology-Distribution Relationships

The importance of morphological traits in bird distribution decreased from 99.36% in the breeding season to 72.28% in the non-breeding season, providing robust evidence that migration plays a dynamic regulatory role in shaping phenotype-habitat associations (Figure 3). This confirms that migration drives niche reallocation through dynamic adjustments in phenotype-environment matching [45]. This phenomenon reflects the morphological matching mechanism during the breeding season and the niche reconstruction effect during the non-breeding season. In the breeding season (Figure 3a), traits such as beak length, tail length, and tarsus length have a strong explanatory power on avian distribution (importance > 99%), consistent with the functional trait environmental filtering theory [57,58]. Specifically, species with long beaks, long tails, and larger body sizes (e.g., Leiothrichidae, Columbidae, and raptors) form dominant clusters at altitudes of 2800–3400 m and 3700–4000 m, with their morphological adaptations matching food acquisition in these habitats. This supports the view that ecological traits influence avian habitat selection [59]. In contrast, species with smaller morphological traits (e.g., Phylloscopidae) are concentrated at 3400–3700 m habitat, likely influenced by the abundant insect resources in this zone [7].

The explanatory power of morphological traits decreases to 72.28% during the non-breeding season (Figure 3b), reflecting the weakening of morphological constraints due to migratory behavior. Omnivorous birds overcome the limitations of morphological traits, such as beak length, by switching diet (insects to fruits) to reselect suitable habitats [59]. Meanwhile, insectivorous birds migrating downward are compressed into the 2800–3100 m zone, occupying the ecological niches vacated by omnivorous birds due to dietary shifts [60]. The seasonal reconstruction of the association between morphology and distribution warns that climate change may disrupt the original phenotypic adaptations [61,62]. This underscores the need for conservation planning to prioritize maintaining habitat heterogeneity in the altitude zones (2800–3400 m), providing refuge for morphologically diverse groups [63].

### 4.3. Seasonal Migration Effects on β-Diversity and Environmental Responses

This study reveals the regulatory role of seasonal migration in avian community assembly mechanisms through a three-dimensional beta diversity partitioning model. During the breeding season, species turnover (contribution rate of 70.44%) and functional turnover (80.02%) dominate diversity changes, a result that is consistent with the study conducted in the central Himalayas [20]. However, this study found that during the non-breeding season, nestedness significantly increased its contribution to both species (49.37%) and functional beta diversity (38.09%). This indicates a seasonal reversal in community composition, where the vertical migration of montane birds leads to changes in species composition, changing the dominance from species and functional turnover to nestedness [64]. Notably, phylogenetic beta diversity is predominantly driven by nestedness throughout the year (52.23% during the breeding season and 53.97% during the non-breeding season), indicating that phylogenetic structure remains largely unaffected by seasonal migration. This finding aligns with global studies on angiosperm beta diversity [65], suggesting that phylogenetic beta diversity is constrained by long-term evolutionary history and niche conservatism, and is less susceptible to short-term environmental fluctuations.

The main environmental factors influencing bird composition are temperature and NDVI (*p* < 0.01) (Figure 5a,b), which is consistent with previous research [27]. Migratory behavior significantly buffers the direct impact of environmental stress on diversity. During the breeding season, the area effect enhances community evenness through habitat heterogeneity (*p* < 0.01), and NDVI is positively correlated with species abundance (*p* < 0.05) and phylogenetic diversity (*p* < 0.05) (Figure 5c), reflecting the productivity-driven niche expansion effect [66]. However, during the non-breeding season, migratory behavior disrupts the direct link between environmental factors and diversity, with the effects of NDVI and temperature on various diversity indices becoming non-significant (*p* > 0.05) (Figure 5d). This suggests that birds mitigate resource competition and low-temperature stress through seasonal vertical migration [67,68]. This seasonal decoupling highlights the key role of migratory behavior in regulating environmental stress, providing a dynamic perspective for the “climate refuge” hypothesis [69]. These findings align with research showing significant seasonal differences in the impact of environmental factors on species diversity [70]. Moreover, this study underscores the critical role of migratory behavior in modulating the impact of environmental factors, emphasizing the importance of dynamic monitoring.

## 5. Conclusions

The results of this study will provide dynamic threshold references for the conservation planning of alpine ecosystems and offer a new paradigm for biodiversity adaptive management under global change. Our study reveals that birds adjust phenotype–environment relationships through seasonal trade-offs in morphological traits, with downslope migrants exhibiting adaptations for flight efficiency and energy savings. Dietary preferences further influence migration strategies, with insectivorous birds tending to migrate downslope, while omnivorous birds exhibit more flexible patterns. Seasonal migration also affects community structure, driving turnover during the breeding season and nestedness during the non-breeding season. Our findings underscore the importance of protecting low-elevation wintering habitats and establishing dynamic protected area networks to support bird conservation in the face of climate change. The study area primarily focuses on the mid-elevation gradient (2800–4000 m) of the Gyirong Valley, which may not fully represent the avian community dynamics in other regions or at higher elevations. Additionally, the time span of data collection is limited. Future research can further explore the seasonal migration patterns of different avian groups over a broader geographical range and how these patterns respond to global climate change. Additionally, conducting long-term monitoring projects to assess the effectiveness of conservation measures and provide data support for the design of dynamic protected area networks will be an important direction for future research.

## Data Availability

The original contributions presented in this study are included in the article. Further inquiries can be directed to the corresponding authors.
